# *Streptococcus Pneumoniae* septic arthritis in adults in Bristol and Bath, United Kingdom, 2006–2018: a 13-year retrospective observational cohort study

**DOI:** 10.1080/22221751.2021.1945955

**Published:** 2021-07-05

**Authors:** Catherine Hyams, Zahin Amin-Chowdhury, Norman K. Fry, Paul North, Adam Finn, Andrew Judge, Shamez N. Ladhani, O. Martin Williams

**Affiliations:** a Academic Respiratory Unit, Learning and Research Building, Southmead Hospital, Bristol, UK; b National Infection Service, Public Health England, London, UK; c Microbiology Services Bristol, Bristol Royal Infirmary, Bristol, UK; d Department of Microbiology, University Hospitals Bristol NHS Foundation Trust, Bristol, UK; e Bristol Children’s Vaccine Centre, Schools of Cellular and Molecular Medicine and of Population Health Sciences, University of Bristol, Bristol, UK; f Musculoskeletal Research Unit, University of Bristol, Bristol, UK

**Keywords:** Pneumococcus, *Streptococcus pneumoniae*, septic arthritis, PCV-13, pneumococcal vaccines, invasive pneumococcal disease

## Abstract

Few studies on adult pneumococcal septic arthritis are sufficiently large enough to assess both epidemiological trends following routine pneumococcal immunization and clinical disease. With major shifts in serotypes causing invasive pneumococcal disease (IPD), we wanted to determine the clinical phenotype of adult septic arthritis caused by *Streptococcus pneumoniae*. We conducted a retrospective cohort study of pneumococcal infections in Bristol and Bath, UK, 2006–2018. We defined pneumococcal septic arthritis as adults with clinically-confirmed septic arthritis, with pneumococcus isolated from sterile-site culture or urinary antigen test positivity. Clinical records were reviewed for each patient in the cohort. Septic arthritis accounted for 1.7% of all IPD cases. 45 cases of adult pneumococcal septic arthritis occurred, with disease typically affecting older adults and those with underlying comorbidity. 67% patients had another focus of infection during their illness. 66% patients required increased care on discharge and 43% had reduced range of movement. In-hospital case fatality rate was 6.7%. One-year patient mortality was 31%. Currently most cases of adult pneumococcal septic arthritis are due to non-PCV13 serotypes which are associated with more severe disease. Non-PCV-13 serotypes had higher prevalence of concomitant pneumococcal infection at another site (73.7% versus 36.6%), increased intensive care or high-dependency unit requirement (32.4% versus 0%), and increased inpatient and 1-year case fatality rate (8.8% versus 0%, and 32.4% versus 27.4% respectively) compared to PCV-13 serotypes. Pneumococcal septic arthritis remains a small proportion of IPD. However, there is significant associated morbidity and mortality, and pneumococcal septic arthritis requires monitoring in coming years.

## Introduction

The annual incidence of septic arthritis in adults in Western Europe ranges between 4 and 10 per 100,000 patient-years and appears to be increasing [1,2]. Septic arthritis is associated with significant morbidity and long-term disability, with case fatality rates (CFR) up to 15% [1,3,4]. Accurate and timely diagnosis is critical for preventing irreversible joint destruction, ankylosis and avascular necrosis [5,6]. Infection can spread locally, damaging bones and nerves, and potentially cause systemic sepsis [6]. Treatment involves empirical antibiotic therapy and removal of the infection source.

*Staphylococcus aureus* is the commonest cause of septic arthritis, causing 30-50% cases. *Streptococcus pneumoniae* is a recognized but rare cause of septic arthritis, causing 2-4% cases, with a higher prevalence in some cohorts [7,8]. Over 100 pneumococcal serotypes are recognized, with the unique capsular polysaccharide of certain serotypes being the antigenic targets in current vaccines. Two vaccines are currently used in the UK. The 23-valent pneumococcal polysaccharide vaccine (PPV23) has been available for over three decades and is indicated for at-risk patients aged ≥2 years and all adults aged ≥65 years [9,10]. The UK implemented the 7-valent pneumococcal conjugate vaccine (PCV7) into the national infant immunization programme in September 2006 [11]. In April 2010, this vaccine was replaced with 13-valent PCV (PCV13) [12]. Both conjugate vaccines have been associated with large declines in vaccine-serotype invasive pneumococcal disease (IPD) across all age groups [11,12], because of direct and indirect (herd) protection. However, increases in IPD due to non-vaccine serotypes (serotype replacement disease) have been reported in the UK [13,14] and other countries [15–18]. In older adults in the UK, serotype replacement disease has nearly cancelled-out the reduction in vaccine-serotype IPD [13]. Consequently, there have been major shifts in pneumococcal serotypes causing IPD, especially in adults and elderly patients, without significant reduction in disease burden.

So far, there have been very few studies on pneumococcal septic arthritis in adults because it is so uncommon. Most studies are too small to assess epidemiological trends following PCV implementation (especially PCV13) or describe clinical characteristics and outcomes of adult patients with septic arthritis due to different serotypes. Therefore, we aimed to describe the epidemiology, serotype distribution, clinical characteristics, risk factors and outcomes of pneumococcal septic arthritis in adults in Bristol and Bath, UK, since PCV7 introduction into the childhood immunization programme in England and eight years after its replacement with PCV13 in 2010.

## Materials and methods

### Study subjects

This study involved adults aged ≥16 years admitted to the three hospitals providing emergency care in Bristol and Bath (University Hospitals Bristol and Weston NHS Trust, North Bristol NHS Trust and The Royal United Hospital) between January 2006 and December 2018. The hospitals and populations are described in Supplementary data 1.

#### Case definitions

Cases were identified retrospectively by searching the Laboratory Information Management System (LIMS) database (Clinisys WinPath Enterprise). *S. pneumoniae* was confirmed on culture of blood or fluid from affected joint(s) at a central laboratory, using standard microbiological techniques outlines in UK standards for microbiology investigations (UK-SMI) combined with API®-20 Strep (BioMérieux, UK) and/or MALDI-TOF (matrix-assisted laser desorption/ionisation/time of flight) mass spectrometer (Bruker, UK). Confirmed cases were linked with the Public Health England (PHE) national reference laboratory to obtain serotype data for each case [19]. Serotyping was performed by phenotypic methods including the Quellung reaction until September 2017, which was replaced by whole genome sequencing thereafter [20]. A positive UAT (BinaxNOW®, Alere, UK) was also considered confirmative of pneumococcal infection. Confirmation of pneumococcal infection in addition to a diagnosis of septic arthritis by the treating physician was considered diagnostic of pneumococcal septic arthritis.

*S. pneumoniae* serotypes contained within each vaccination are listed in Supplementary data 2.

#### Data collection

Detailed clinical records were obtained from each hospital and were reviewed for the following data: age, gender, smoking status, co-morbidities, concomitant pneumococcal infection, joint(s) affected, treatment including surgical management, and clinical outcomes. Laboratory findings were examined in addition to clinical observations on presentation of the septic arthritis episode.

#### Data analysis and statistics

Data were analysed using SPSS, version 25.0 (New York, IBM). Incidence rates were calculated by dividing number of cases by the adult population within the catchment area of the three hospitals. Categorical variables are presented as counts and percentiles. Continuous data were tested for normality using the Shapiro–Wilk Test, with *P*<0.05 considered significant: normally distributed data are presented as means and standard deviations (SD), whilst data that did not follow a normal distribution are presented as medians with interquartile ranges (IQR). Non-parametric data were analysed using appropriate statistical tests including the two-sided Chi-squared test.

#### Ethics approval

The study was approved by the Health Research Authority, UK (IRAS 265437).

## Results

### Patient cohort and epidemiology

**Figure 1.**
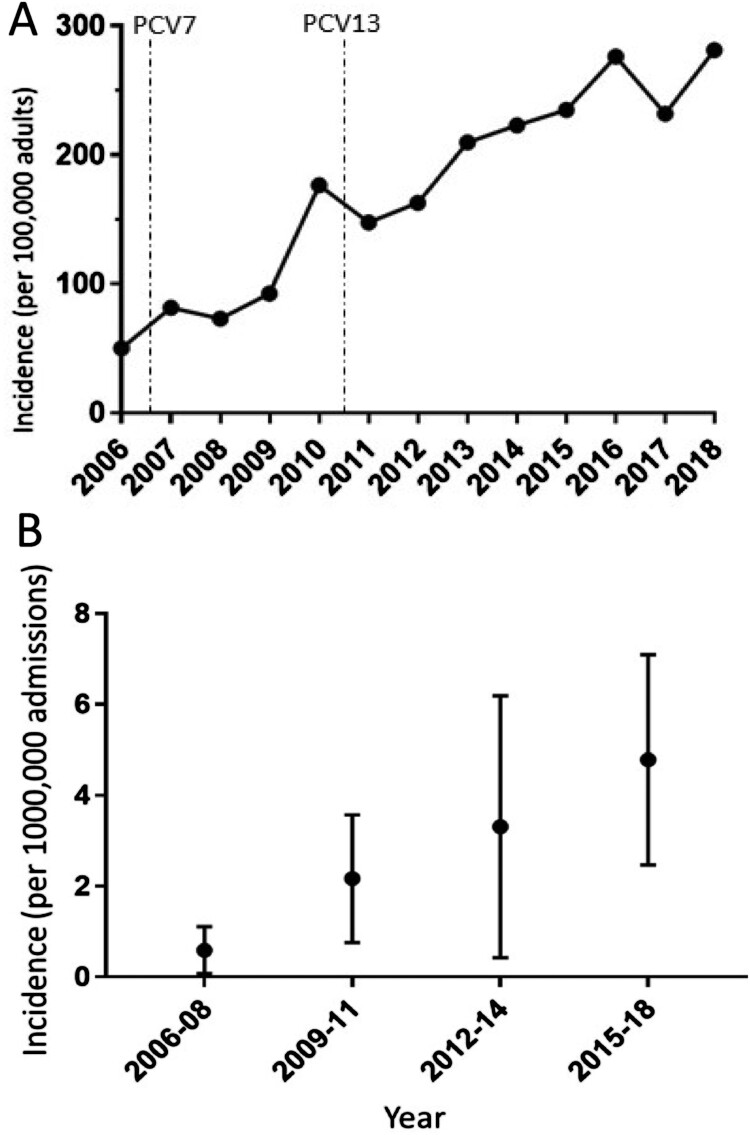
**Adult hospital admissions attributable to *Streptococcus pneumoniae****.* Rate of disease for hospitalizations due to (A) all pneumococcal disease and (B) septic arthritis per 100,000 adults. Error bars represent 95% CI. The dashed lines in Figure 1(A) show the introduction of PCV7 and PCV13. Disease incidence was calculated using adult population data from the Office of National Statistics (Supplementary data 1).

We identified 2657 clinical episodes of IPD between January 2006 and December 2018: 45 (1.7%) had septic arthritis (Table 1). The median age of patients with septic arthritis was 68 years (IQR 56-78, range 34-92). IPD requiring hospitalization increased throughout the study period (Figure 1A) and incidence of pneumococcal septic arthritis requiring hospitalization also increased (Figure 1B). Annual blood and joint cultures increased by 38% and 23% respectively from 2006 to 2018, with the increase in testing less than the increase in pneumococcal septic arthritis detected in hospitalized patients.

**Table 1.** Characteristics of patients with *S. pneumoniae* septic arthritis between 2006 and 2018.

Characteristic or finding	All patients n (%)
*Age (years)*	
16-29	1 (2.2)
30-49	6 (13.3)
50-64	9 (20.0)
65-79	19 (42.2)
>80	10 (22.2)
*Gender*	
Male	20 (44.4)
Female	25 (55.6)
*Joint(s) involved*	
Hip	1 (2.2)
Knee	23 (51.1)
Ankle	6 (13.3)
Shoulder	11 (24.4)
Elbow	5 (11.1)
Wrist	5 (11.1)
Toe	2 (4.4)
Prosthetic joint	5 (11.1)
Monoarticular	37 (82.2)
Polyarticular	8 (17.8)
*Other Infection Sites*	
Preceding URTI	14 (31.1)
Concomitant infection	30 (66.6)
Pneumonia	26 (57.8)
Meningitis	3 (6.7)
Other	1 (2.2)
*Risk Factors*	
Recent blunt trauma to joint	16 (35.6)
Underlying disease/risk factors	43 (95.6)
*Clinical presentation*	
Red, hot, swollen joint	43 (95.6)
Temperature ≥38°C	35 (77.8)
Tachycardia (heart rate ≥100bpm)	20 (44.4)
Hypotension †	6 (13.3)
Confusion (AMTS ≤7)	7 (15.6)
Biochemistry Investigations	median (IQR)
- Sodium (mmol/L)	135 (133-141)
- Creatinine (μmol/L)	111 (78-134)
- Urea (mmol/L)	8.1 (5.3-10.7)
- eGFR (mL/min/1.73m^2^)	57 (38-70)
- C-reactive protein (mg/L)	213 (126-307)
- Albumin (g/L)	30.0 (27.0-32.0)
Haematology Investigations	median (IQR)
- WBC (×10^9^/L)	15.0 (12.2-20.1)
- Neutrophil (×10^9^/L)	13.1 (10.2-16.9)
- Lymphocyte (×10^9^/L)	1.3 (0.9-2.1)
Microbiological diagnosis	n/total n (%)
- Blood culture positive	28/45 (62.2)
- UAT positive	8/45 (17.8)
- Fluid culture positive only	13/45 (28.9)
Positive joint fluid *S. pneumoniae* results	n/total n (%)
- Gram stain	37/45 (82.2)
- Culture	43/45 (95.6)
- White cell count - average (range)	112,000 cells/mm^3^ (5,900–302,000 cells/mm^3^)

AMTS, abbreviated mental test score; DBP, diastolic blood pressure; eGRF, estimated Glomerular Filtration Rate; SBP, systolic blood pressure; UAT, urinary antigen test; URTI, upper respiratory tract infection; WBC, white blood cell

Normal ranges for haematology were white blood cell count (WBC) 4.0-11.0×10^9^/L, neutrophils 1.5-8.0×10^9^/L and lymphocytes 1.25×10^9^/L.

† Hypotension was defined as SB*P*<100mmHg or DBP ≤60mmHg.

### Clinical characteristics

Typical presentation of pneumococcal septic arthritis was sudden onset joint pain with a swollen, hot joint and associated fever (≥38°C). Tachycardia occurred in 44% patients, but hypotension was uncommon. Symptoms were present for median 3 days (IQR1-4, range 0-5) before diagnosis for those who survived, and 4 days (IQR2-5, range 2-5) in non-surviving patients (Table 1).

Thirty patients (67%) had pneumococcal infection at a second site, including pneumonia in 26 (86% of second site cases) (with one case complicated by empyema); two cases (7% second site cases) of meningitis and one of pneumonia and meningitis; and one case of mycotic aortic aneurysm. For most (93%) patients in this study, the admitting physician considered septic arthritis the primary infection focus. Most patients (82%) had monoarticular septic arthritis: the knee was the most common joint involved (23 joints, 51%), followed by the shoulder (11 joints, 24%). There were five prosthetic joint infections: four knee and one hip infection.

### Underlying disease(s)/Risk factors

Most patients (96%) had more than one co-morbidity or risk factor (including recreational drug use, joint trauma, severe malnutrition) predisposing them to disease. One patient was pregnant, sustained trauma to the affected joint and subsequently underwent early Caesarean section in the third trimester with good outcome. Medical co-morbidities occurring frequently included hypertension (*n *= 14), diabetes mellitus (*n *= 9), chronic renal failure (*n *= 4), left ventricular failure (*n *= 5) (Table 1).

Pre-existing malignancies included renal cell carcinoma (2); malignant melanoma (2); colon (1), oesophageal (1), and breast cancer (1). Two patients were diagnosed with multiple myeloma during admission for pneumococcal septic arthritis. Pre-existing joint disease was documented in 19 patients, including osteoarthritis and rheumatoid arthritis. Smoking, alcohol misuse and injecting drug use (IDU) was reported in 24, 5 and 2 patients, respectively.

Twenty nine patients were aged ≥65 years, and a further 7 deemed eligible for PPV23 vaccination under current UK guidelines [9]; 20 of these individuals had received PPV23 vaccination prior to their pneumococcal septic arthritis.

### Outcome

The median length of hospital admission was 25 days (IQR 16-30, range 7-62) with two non-survivors dying <72 h from admission and 25 survivors discharged home. 10 patients transferred to a rehabilitation/specialist centre. Almost half of survivors (47%) required additional care on discharge (Table 2).

**Table 2.** Treatment and outcomes of *S. pneumoniae* septic arthritis.

Treatment or outcome	Cohort patients
Antibiotic agents	
Penicillin	31/45 (68.9)
- alone	13/45 (28.9)
- in combination *	18/45 (40.0)
Cephalosporin	12/45 (26.7)
Fluoroquinolone	2/45 (4.4)
Antibiotic treatment	
Single antibiotic class	28/45 (62.2)
Multiple antibiotic classes	17/45 (37.8)
Intravenous	
- initial mode of administration	44/45 (97.8)
- unable to rationalize to oral	5/45 (11.1)
Days until rationalization – average ± SD	16.4 ± 6.5
Length of antibiotic course – average ± SD	67.2 ± 14.1
Surgical Management & 12 month outcomes	
Arthroscopic drainage	35/45 (77.8)
- Reduced ROM	15/35
- Cured	12/35
- Unknown joint function	8/35
Incision and drainage	8/45 (17.8)
- Reduced ROM	2/8
- Cured	3/8
- Unknown joint function	3/8
Complications	
ITU/HDU care**	11/45 (24.4)
- I&V	10/45 (22.2)
- Inotropic support	8/45 (17.8)
Acute renal failure	9/45 (20.0)
Liver dysfunction	4/45 (8.9)
Admission	
Hospital length of stay – average (± SD)	24.9 ± 14.2
Discharge destination	
- Home	25/42 (59.5)
- Rehab/specialist centre	10/42 (23.8)
- Increased care requirement†	20/42 (47.6)
Case fatality rate	
Inpatient	3/45 (6.7)
- Monoarticular infection	1/45 (2.2)
- Polyarticular infection	2/45 (4.4)
1-year mortality	14/45 (31.1)

HDU, high dependency unit; I&V, intubation and ventilation; ITU, intensive therapy unit; ROM, range of movement.

*Penicillin class antibiotic (including Benzylpenicillin, amoxicillin, co-amoxiclav, flucloxacillin) was given in combination with an aminoglycoside (*n *= 1), cephalosporin (*n *= 1), glycopeptide (*n *= 14), macrolide (*n *= 1), and nitroimidazole and aminoglycoside (*n *= 1).

**ITU/HDU care in addition to any requirement in the post-operative recovery period.

†Increased care requirement includes increasing packages of care or other support for patients discharged home, discharge to new care facility from patient’s home or increasing care facility requirement (eg nursing home from assisted living).

Three (7%) patients died during hospital admission: all had severe comorbidities. One had malignant melanoma, diabetes and ischaemic heart disease and was the only patient with WBC<4.0×10^9^/L at presentation. The second had a history of alcohol misuse, malnutrition and sustained significant joint trauma; the third patient had malignant melanoma, ischaemic heart disease, cerebrovascular disease and dementia. All these patients had a lymphocyte count<1.0×10^9^/L on admission (although eleven other patients with lymphopenia survived). Two of these patients had polyarticular infection with concomitant pneumonia; all had infection with a PPV23 serotype not in PCV13 (PPV23nonPCV13) (8, 12F and 33F). Patients with cancer had poor survival with four fatalities within 1 year of presentation (CFR 44%): two died during hospital admission and two after discharge.

Ten additional patients died within one year of presentation (overall one-year fatality, 31%). Six patients succumbed to respiratory infection, with no causative pathogens identified. Of the 31 patients surviving over a year, 28 had follow-up records detailing recovery: 15 recovered joint function, and 13 had reduced range of movement (ROM). Three patients with osteoarthritis recovered baseline joint function. All patients with complicated pneumococcal infection (empyema, meningitis or mycotic aneurysm) had long-term reduced ROM or died.

#### Investigation findings

Initial investigation results are summarized in Table 1. 37 patients had leucocytosis (defined as WBC >11.0×10^9^/L) and 42 had neutrophilia (defined as neutrophils >7.7 × 10^9^/ L). One patient had leukopaenia (defined as WBC <3.0×10^9^/L) and 14 patients had lymphopenia (defined as lymphocytes <1.5 × 10^9^/L). C-reactive protein was >300 mg/L in 12 patients (one of whom died), between 200 and 300 mg/L in 14 patients (two of whom died) and 100-200 mg/L in 19 patients. Most patients were hypoalbuminaemic: 26 patients (57%) had albumin ≤30 g/L (normal range 35-50 g/L).

All patients had joint cultures taken: 43 (96%) were positive for pneumococcus. Both patients with negative joint cultures had fluid obtained >24 h after antibiotic commencement. 13 (29%) patients were diagnosed through positive joint culture alone. Blood cultures were positive in 28 patients, while 8 had a positive BinaxNOW® UAT; notably, two patients had a positive UAT but negative sterile-site cultures (Table 1).

#### Management

Most patients (62%) received intravenous flucloxacillin and another penicillin. Following confirmation of pneumococcal infection, antimicrobial prescribing was amended in 23 (51%) patients, most commonly to benzylpenicillin, ceftriaxone or cefuroxime. The most utilized single-agent antimicrobial was a penicillin (Table 2). In the 42 surviving adults, mean duration of intravenous therapy was 16.4 ± 6.5 days, and total length of course was 67.2 ± 14.1 days, with six adults receiving 90 d’s therapy. In total, 11 (24%) patients required treatment on intensive care (ITU) or a high-dependency unit (HDU), most frequently for ventilatory support necessitated by second-site pneumonia; all patients with concomitant meningitis required ITU/HDU care.

Arthroscopic drainage was performed in 35 patients, and eight others underwent open incision and drainage. Three patients requiring open surgery had polyarticular infection and the affected joints included the knee (*n *= 6), ankle (*n *= 2) and shoulder (*n *= 3).

### S. pneumoniae strains and outcomes

**Figure 2.**
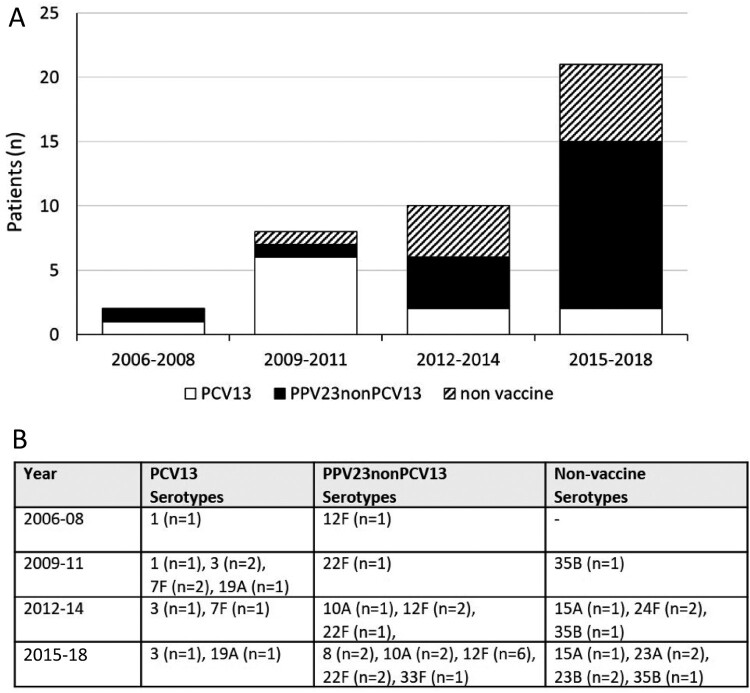
**Pneumococcal septic arthritis by vaccine group.** Figure 2(A) Cases of pneumococcal septic arthritis by vaccine group. White represents disease from serotypes contained within PCV13, black represents disease from PPV23nonPCV13 serotypes and dashed lines represents disease from serotypes not contained in current pneumococcal vaccines. Figure 2(B) The number of cases attributable to individual serotypes, by vaccine type*, are listed within the table. * Serotypes included in both PCV13 and PPV23 are 1, 3, 4, 5, 6B, 7F, 9 V, 14, 18C, 19F, 19A, and 23F. Additional serotypes in PPV23 are 2, 8, 9N, 10A, 11A, 12F, 15B, 17F, 20, 22F, 23F, and 33F, whereas serotype 6A is only included in PCV13 but not PPV23. PCV7 included serotype 4, 6B, 9 V, 14, 18C, 19F and 23F which are also included in both PCV13 and PPV23.

Serotype data were available for 39 patients (87%). The most commonly isolated serotype was 12F (*n *= 9), followed by 22F (*n *= 4) and 3 (*n *= 4). The proportion of cases due to PCV13 serotypes increased after PCV7 implementation in 2006, plateaued after PCV13 implementation in 2010 before declining in recent years. During the last 6 study years (2013-2018), the majority of cases (70%) were due to serotypes included in PPV23 only with eight cases due to non-vaccine serotypes (Figure 2).

Of the 11 cases due to PCV13 serotype (Table 3) none required HDU/ITU care. These patients survived hospital admission: three died within one year, five survivors had reduced ROM, and four required increased care after hospital discharge.

**Table 3.** *S. pneumoniae* septic arthritis features by pneumococcal vaccine type.

Characteristic or finding	PCV13 serotype *n *= 11	PPV23nonPCV13 serotype *n *= 19	Non-Vaccine serotype *n* = 11	Other *n *= 4	Total *n *= 45
Male gender – n (%)	5 (45.5)	8 (42.1)	5 (45.5)	2 (50.0)	20 (44.4)
Age (y) – median (IQR)	66 (59-76)	67 (58-77)	68 (53-79)	69 (62-74)	68 (56-78)
Smoker – n (%)	3 (27.3)	5 (26.3)	4 (36.4)	2 (50.0)	14 (31.1)
*Joint(s) involved – n (%)*					
Knee	6 (54.5)	9 (47.4)	5 (45.5)	3 (75.0)	23 (51.1)
Ankle	2 (18.2)	2 (10.5)	2 (18.2)	0 (0.0)	6 (13.3)
Shoulder	2 (18.2)	4 (28.6)	3 (27.2)	2 (50.0)	11 (24.4)
Elbow	1 (9.1)	2 (10.5)	1 (9.1)	1 (20.0)	5 (11.1)
Wrist	1 (9.1)	2 (14.3)	1 (9.1)	1 (20.0)	5 (11.1)
Prosthetic joint	1 (9.1)	2 (10.5)	1 (9.1)	0 (0.0)	5 (11.1)
Monoarticular	9 (81.8)	15 (78.7)	10 (90.9)	3 (75.0)	37 (82.2)
Polyarticular	2 (18.2)	4 (21.1)	1 (9.1)	1 (25.0)	8 (17.8)
*Second Site Infection*					
Second site infection	4 (36.6)	16 (84.2) *	7 (63.3) *	2 (50.0)	30 (66.6)
Pneumonia	4 (36.6)	14 (73.7) *	7 (63.3) *	2 (50.0)	26 (57.8)
Meningitis	0 (0.0)	3 (15.8)	0 (0.0)	0 (0.0)	3 (6.7)
Other	0 (0.0)	1 (5.3)	0 (0.0)	0 (0.0)	1 (2.2)
*Complications – n (%)*					
ITU/HDU care**	0 (0.0)	7 (36.8) *	2 (18.2)	2 (50.0)	11 (24.4)
- I&V	0 (0.0)	6 (54.5) *	2 (18.2)	2 (50.0)	10 (22.2)
- Ionotropic support	0 (0.0)	4 (21.1) *	2 (18.2)	2 (50.0)	8 (17.8)
Acute renal failure	3 (27.3)	4 (21.1)	0 (0.0)	2 (50.0)	9 (20.0)
*Admission – n (%)*					
Hospital length of stay – average ± SD	25.0 ± 10.9	28.2 ± 14.9	27.4 ± 13.6	25.9 ± 16.1	24.9 ± 14.2
Discharge destination					
- Home	6 (54.5)	10 (52.6)	6 (54.5)	3 (75.0)	25 (55.6)
- Rehab/specialist centre	3 (27.3)	3 (21.4)	2 (18.2)	2 (50.0)	10 (22.2)
- Increased care need	4 (36.6)	8 (42.1)	6 (54.5)	2 (50.0)	20 (44.4)
*Joint Function – n (%)*					
Reduced ROM	5/8 (62.5)	6/16 (37.5)	3/8 (37.5)	1/3 (33.3)	15/35 (42.9)
Cured	3/8 (37.5)	4/16 (25.0)	4/8 (50.0)	1/3 (33.3)	12/35 (34.3)
*Case fatality rates – n (%)*					
Inpatient	0 (0.0)	3 (21.4) *	0 (0.0)	0 (0.0)	3 (6.7)
1-year mortality	3 (27.3)	5 (26.3)	4 (36.4)	2 (50.0)	14 (31.1)

HDU, High dependency unit; I&V, intubation and ventilation; ITU, intensive therapy unit; ROM, range of movement.

* *P*<0.01 Chi-squared test compared to PCV13 serotype disease.

** ITU/HDU care in addition to any requirement in the post-operative recovery period.

PCV13 disease comprised of serotypes: 1 (*n *= 2), 3 (*n *= 4), 7F (*n *= 3) and 19A (*n *= 2); PPV23nonPCV13 disease comprised serotypes: 8 (*n *= 2), 10A (*n *= 3), 12F (*n *= 9), 22F (*n *= 4), 33F (*n *= 1); Non-vaccine serotypes included: 15A (*n *= 2), 23A (*n *= 2), 23B (*n *= 2), 24F (*n *= 2), 35B (*n *= 3). Other disease includes lost serotype or only UAT positive disease.

Serotypes in PPV23 only were responsible for 19 cases: concomitant infection at another site was more frequent in this group compared to those with PCV13 serotype disease (*P *< 0.05). Fourteen had pneumonia and all cases of septic arthritis associated with meningitis were attributable to serotypes 12F and 22F. Seven required ITU/HDU care; notably, this group accounted for 64% of patients requiring HDU/ITU care. Inpatient CFR but not 1-year CFR was higher compared to those with PCV13 disease (*P *< 0.05).

Non-vaccine serotypes were responsible for 11 cases, and none died of septic arthritis. Seven survived over a year.

## Discussion

We have described one of the largest cohorts of patients with pneumococcal septic arthritis occurring over 13 years in South West England. This is the first report describing epidemiological trends of adult pneumococcal septic arthritis, including clinical characteristics and patient outcomes, following paediatric PCV implementation in the UK (PCV7 and PCV13). Septic arthritis accounted for 1.3% of IPD cases and many patients were elderly and/or had underlying comorbidities. A high proportion of patients had pneumococcal infection at a second site, mainly pneumonia, with three cases of meningitis. There was a trend towards increasing incidence of pneumococcal septic arthritis after polysaccharide conjugate vaccination (PCV7) introduction. Consistent with this observation was an increase followed by a decrease in pneumococcal septic arthritis cases caused by the additional PCV13 serotypes not included in PCV7. Pneumococcal septic arthritis was associated with an in-hospital case fatality rate of 6.7% and 31% died within 12 months, demonstrating similar fatality rates to hip fractures [21].

### Disease

Prior to the UK routine implementation of PCVs, septic arthritis represented 2.4% of IPD cases compared to 1.3% since PCV7 introduction [7]. This is consistent with 1.5% reported in recent studies from France and Canada [22,23]. Patients with pneumococcal septic arthritis in our cohort were typically elderly, with two or more risk factors pre-disposing them to IPD. A case series and literature review of 108 adults reported mean age 59 years [8], while 80% cases diagnosed during 1985–1998 in Nottingham, UK, were aged >60 years [7] consistent with more recent studies [22,24]. Additionally, 79-92% patients with pneumococcal septic arthritis in reported studies had recognized risk factors. In a case series of 51 patients from Alberta, Canada, an inability to walk independently, male gender and underlying joint disease were identified as important risk factors for pneumococcal septic arthritis [23]. Uniquely, one patient in our cohort was pregnant when she developed pneumococcal septic arthritis; this has not been reported previously in the literature.

In keeping with published studies, the knee was the most affected joint: this included prosthetic joint infection, which contributed 11% to our cohort of cases. This prevalence is at the lower end of the range reported (11-31%) [7,8,22,24] and may arise from factors unaccounted for by this study, including the number of prosthetic implants performed in our area. *S. pneumoniae* should be considered as a potential cause of septic arthritis in elderly patients presenting with swollen joints, especially knee and shoulder joints.

Over half (58%) our cohort had concomitant pneumococcal infections at another site, mainly pneumonia, at the higher end of the 28-56% range reported by other studies [7,8,24]. Septic arthritis was considered the primary focus of infection in almost all the patients in this cohort, with joint pain or loss of function being reported as the main presenting symptom’s the second site infection was detected on further clinical examination and investigation. This highlights the importance of thorough clinical examination – especially of the joints – in elderly patients presenting with features of pneumonia, which was frequently the reason for such patients to require HDU/ITU care.

### Deaths and outcomes

In keeping with previous studies and with septic arthritis overall, the in-hospital CFR for pneumococcal septic arthritis in our cohort was low (6.7%). The clinical team noted that all patients who died were frail. Two died within 72 h of admission; all developed respiratory failure secondary to severe bilateral pneumonia. However, by one year after hospitalization, 31% patients had died, including 80% of patients aged ≥80 years. Further studies are needed to identify potentially modifiable factors to improve long-term outcomes of pneumococcal septic arthritis in the elderly.

Of those who survived, 22% patients required discharge to a rehabilitation or specialist treatment unit. Additionally, 42% (*n *= 13) had long-term reduced ROM, substantially higher than the 21-36% reported in the literature [7,8,22]. All patients with complicated pneumococcal infection (empyema, meningitis or mycotic aneurysm) had long-term reduced ROM of the affected joint.

The increased care requirement at discharge was particularly common for older patients who frequently required additional mobility aids and care. Knowledge of functional loss and additional care need is important for setting the expectations of patients and their families in respect to future care requirement. Furthermore, the arrangement of care packages and equipment often results in delays in hospital discharge [25], increasing the risk of hospital-acquired infection and decline in mobility and loss of independence for patients, as 5% of muscle strength is lost with each day in hospital [26–28].

### Implications

The epidemiology of pneumococcal serotypes causing septic arthritis has shifted over the past decade due to the implementation of two pneumococcal immunization programmes: PCV7 in 2006 and PCV13 in 2010. Others have reported increasing incidence of pneumococcal septic arthritis following PCV13 introduction, with 40% of cases attributable to non-PCV13 serotypes [22]. We found differences in disease presentation between the different vaccine serotype groups, particularly septic arthritis due to PPV23nonPCV13 and non-vaccine serotypes, which appeared to be associated with more severe disease, including a higher prevalence of concomitant pneumococcal infection at another site, increased ITU/HDU admission, and higher inpatient CFR in comparison to PCV13 serotype adult septic arthritis. Numbers of cases, however, were small and, therefore, additional studies are required to confirm this finding, especially given that currently nearly all cases are due to non-PCV13 and non-vaccine serotypes in the UK [13].

### Strengths and limitations

This study captured patients with proven pneumococcal disease in a large geographical area with a population of nearly a million adults, encompassing 3 large hospitals with 100000 unplanned admissions and 270000 emergency department attendances by adult patients annually. We studied pneumococcal disease over 13 years, spanning the period before and after PCV13 implementation, allowing us to assess disease trends over time. Furthermore, linking our cases with the PHE national reference laboratory provided serotype information for almost all cases. A major strength is that epidemiological data were supported with detailed clinical information for individual patients, including short- and long-term outcomes.

This is a retrospective observational study; therefore, only data documented in clinical records could be collected. Despite the large IPD cohort, pneumococcal septic arthritis was diagnosed in only a small proportion of cases. Additionally, our regional findings may not apply to other populations. Patients were managed according to clinicians’ discretion, which may have influenced the investigation and management of individual patients, including requirement for HDU/ITU care, surgical management and discharge preferences. We included patients who tested positive for pneumococcus using the BinaxNOW® urinary antigen test, with a known sensitivity of 65% and prolonged positive test result after exposure to pneumococcus [29], although only two patients were solely diagnosed in this way. Finally, different methodologies were used during the study period to identify pneumococcal serotypes; whole genomic sequencing was not routinely used by PHE until October 2017, and therefore most isolates did not have this performed.

In conclusion, pneumococcal septic arthritis is responsible for a small proportion of IPD cases and mainly affected the elderly, who often have multiple underlying comorbidities. While inpatient CFR was low, almost a third of patients died within one year. The increasing rate and severity of pneumococcal septic arthritis, currently mainly due to non-PCV13 serotypes, requires close monitoring in coming years.

## Declaration of interest statement

CH is Principal Investigator of the Avon CAP study which is an investigator-led University of Bristol study funded by Pfizer and has previously received support from the NIHR in an Academic Clinical Fellowship. AF is a member of the Joint Committee on Vaccination and Immunization (JCVI) and chair of the World Health Organization European Technical Advisory Group of Experts on Immunization (ETAGE) committee. In addition to receiving funding from Pfizer as Chief Investigator of this study, he leads another project investigating transmission of respiratory bacteria in families jointly funded by Pfizer and the Gates Foundation. The other authors have no relevant conflicts of interest to declare.

## Supplementary Material

Supplemental Material
