# Supplementary material for: Streptococcus Pneumoniae septic arthritis in adults in Bristol and Bath, United Kingdom, 2006–2018: a 13-year retrospective observational cohort study
Source: Emerg Microbes Infect. 2021 Jul 5;10(1):1369–77. doi: 10.1080/22221751.2021.1945955 (PMC8259820; doi:10.1080/22221751.2021.1945955)
Supplement: Supplemental Material [file TEMI_A_1945955_SM2777.docx]

**Supplementary Table 1: Annual Population and Hospital Admissions**

Population size within the Bristol, North Somerset and South Gloucestershire CCG and Bath & NE Somerset CCG as based on data obtained from the Office for National Statistics (ONS).

Number of adult patients seen are listed as a combined total for all 3 NHS Trusts, from data provided by NHS Digital. Data for patients seen in Emergency Departments is derived from Hospital Accident and Emergency Activity. Data for admissions is collated from Monthly Hospital Episode Statistics for Admitted Patient Care, Outpatient and Accident and Emergency data

|  | 2006 | 2007 | 2008 | 2009 | 2010 | 2011 | 2012 | 2013 | 2014 | 2015 | 2016 | 2017 | 2018 |
| --- | --- | --- | --- | --- | --- | --- | --- | --- | --- | --- | --- | --- | --- |
| Population (n) |  |  |  |  |  |  |  |  |  |  |  |  |  |
| **Whole population** | 1,031,918 | 1,040,674 | 1,047,210 | 1,053,791 | 1,028,596 | 1,070,120 | 1,080,886 | 1,131,336 | 1,104,211 | 1,118,820 | 1,131,336 | 1,139,791 | 1,140,236 |
| **≥ 16 y** | 823,298 | 833,548 | 839,310 | 844,186 | 866,522 | 887,470 | 925,556 | 893,556 | 902,950 | 915,438 | 925,566 | 933,304 | 940,293 |
| **16-34 y** | 237,508 | 287,368 | 288,798 | 288,536 | 297,243 | 306,361 | 301,317 | 301,317 | 305,932 | 313,489 | 319,384 | 322,914 | 323,715 |
| **35-49 y** | 222,704 | 224,970 | 225,290 | 225,944 | 227,259 | 220,217 | 219,883 | 219,883 | 217,895 | 217,136 | 216,015 | 215,618 | 215,987 |
| **50-64 y** | 176,998 | 178,690 | 179,822 | 180,714 | 184,550 | 186,294 | 185,960 | 185,960 | 188,442 | 191,215 | 194,082 | 196,991 | 197,565 |
| **65-79 y** | 106,488 | 107,636 | 109,918 | 112,516 | 118,948 | 129,021 | 140,629 | 133,018 | 136,428 | 138,803 | 140,629 | 141,792 | 142,895 |
| **≥80 y** | 34,094 | 34,884 | 35,487 | 36,476 | 38,522 | 45,577 | 55,456 | 53,378 | 54,253 | 54,795 | 55,456 | 55,989 | 60,131 |
| Adult admissions (n) |  |  |  |  |  |  |  |  |  |  |  |  |  |
| **Accident and Emergency** | 266,411 | 261,328 | 270,012 | 266,714 | 268,253 | 271,948 | 279,953 | 286,314 | 275,960 | 28,3137 | 263,566 | 269,868 | 271,328 |
| **Unplanned admissions** | 103,590 | 96,665 | 123,004 | 105,774 | 108,151 | 107,704 | 106,753 | 109,235 | 114,415 | 120,960 | 126,315 | 133,640 | 137,521 |

**Supplementary Table 2: Serotypes contained within each pneumococcal vaccination.**

| **Vaccination** | **Serotypes** |
| --- | --- |
| Pneumococcal polysaccharide vaccine, 23 valent  (PPV-23, PneumoVax®) | 1, 2, 3, 4, 5, 6B, 7F, 8, 9N, 9V, 10A, 11A, 12F, 14, 15B, 17F, 18C, 19F, 19A, 20, 22F, 23F, 33F. |
| Pneumococcal conjugate vaccine, 7-valent (PCV-7, Prevenar®) | 4, 6B, 9V, 14, 18C, 19F and 23F |
| Pneumococcal conjugate vaccine, 13-valent (PCV-13, Prevenar13®) | 1, 3, 4, 5, 6A, 6B, 7F, 9V, 14, 19A, 19F, 18C, and 23F |

**Supplementary Data: NHS Trusts**

There are three hospitals in the Bristol and Bath area: The Bristol Royal Infirmary (University Hospitals Bristol NHS Foundation Trust), Southmead Hospital (North Bristol NHS Trust), and The Royal United Hospital in Bath. These hospitals are summarised in the table beneath:

**Summary of Hospital and Services Provided:**

|  | **Bristol Royal Infirmary** | **Southmead Hospital** | **Royal United Hospital** |
| --- | --- | --- | --- |
| Location | Central Bristol | North Bristol | Bath |
| **Hospital Capacity (beds**) ^1^ | |  |  |
| Total | 821 | 929 | 759 |
| Medical | 440 | 538 | 423 |
| Surgical | 178 | 274 | 117 |
| Critical Care (ICU/HDU) | 39 | 44 | 13 |
| **Specialist Services** |  |  |  |
| Specialist Services | Cystic Fibrosis | Neurosciences | Rheumatology |
| (adult) | Cardio-thoracic | Orthopaedic Centre |  |
|  | Haem & Oncology | Plastics/Reconstruction |  |
|  | Ophthalmology | Major Trauma |  |
|  | Ear, Nose & Throat Surgery | Renal & Transplant |  |
|  | Oral & Maxillofacial Surgery | Urology |  |
|  | Dental Services | Vascular Services |  |
|  | Sleep & ventilation | Pleural Medicine |  |

**^1^** Data extracted from NHS England: SDCS data collection- KH03, available at https://www.england.nhs.uk/statistics/statistical-work-areas/bed-availability-and-occupancy/bed-data-overnight/
